# Association of vancomycin trough concentration on the treatment outcome of patients with bacteremia caused by *Enterococcus* species

**DOI:** 10.1186/s12879-021-06809-x

**Published:** 2021-10-26

**Authors:** Yujin Sohn, John Hoon Rim, Yunsuk Cho, Jonghoon Hyun, Yaejee Baek, Moohyun Kim, Jung Ho Kim, Hye Seong, Jin Young Ahn, Sang-Guk Lee, Jong-Beack Lim, Su Jin Jeong, Nam Su Ku, Jun Yong Choi, Joon-Sup Yeom, Young Goo Song

**Affiliations:** 1 Department of Internal Medicine, Yonsei University College of Medicine, 50-1 Yonsei-ro, Seodaemun-gu, Seoul, 03722 Republic of Korea; 2 AIDS Research Institute, Yonsei University College of Medicine, Seoul, South Korea; 3 Department of Laboratory Medicine, Yonsei University College of Medicine, 50-1 Yonsei-ro, Seodaemun-gu, Seoul, 03722 Republic of Korea

**Keywords:** Vancomycin, *Enterococcus*, Trough level, AUC/MIC

## Abstract

**Background:**

Pharmacokinetic-pharmacodynamic (PK/PD) targets of vancomycin therapy have been recognized for methicillin-resistant *Staphylococcus aureus* infections but not for other gram-positive bacterial infections. Therefore, we investigated whether vancomycin concentration targets such as the trough level and ratio of the area under the curve to minimum inhibitory concentration (AUC/MIC) are associated with the treatment outcome in enterococcal bacteremia.

**Methods:**

A retrospective cohort analysis enrolled patients with bacteremia caused by vancomycin-susceptible *Enterococcus faecium* and *Enterococcus faecalis* who were treated with vancomycin from January 2007 to December 2017 at a tertiary hospital located in Seoul, South Korea. Patients without vancomycin concentrations were excluded from the study. The primary outcome was 28-day all-cause mortality.

**Results:**

A total of 37 patients were enrolled—26 with *E*. *faecium* infection and 11 with *E*. *faecalis* infection. The 28-day all-cause mortality rate was 21.6 %. In univariate analysis, vancomycin trough level (≤ 15 µg/mL; *p* = 0.042), age (*p* = 0.044), and septic shock (*p* = 0.049) were associated with 28-day mortality but not AUC24/MIC (> 389; *p* = 0.479). In multivariate analysis, vancomycin trough concentration (≤ 15 µg/mL; *p* = 0.041) and younger age (*p* = 0.031) were associated with 28-day mortality in patients with enterococcal bacteremia.

**Conclusions:**

In this study, a vancomycin trough level of 15 µg/mL or lower was associated with 28-day mortality in enterococcal bacteremia. However, relatively large prospective studies are needed to examine the efficacy of vancomycin PK/PD parameters in patients with enterococcal bacteremia.

**Supplementary Information:**

The online version contains supplementary material available at 10.1186/s12879-021-06809-x.

## Background

*Enterococcus* species are ubiquitous in natural environments, including plants, soil, and water, and are part of the normal gastrointestinal flora of humans and other animals. Their broad distribution allows them to survive and persist in various environments [1]. Because of their ability to colonize medical devices and their high viability in nosocomial environments, *Enterococcus* species are a major cause of hospital-acquired infections [2]. Enterococci are important nosocomial pathogens worldwide, accounting for 14% of all hospital-acquired infections in the United States between 2011 and 2014 [3]. *Enterococcus* species cause infections of the bloodstream, urinary tract, and surgical sites, *inter alia*. These infections can lead to infective endocarditis, urinary tract infections, bacteremia, peritonitis, and prosthetic joint infections. In many centers, the proportion of ampicillin-resistant *Enterococcus faecium* exceeds 70%. *Enterococcus faecalis* resistant to ampicillin is rare, but resistance rate is increasing in nosocomial infections and the rate of ampicillin-resistant *E. faecalis* is reported to be 1.8%; hence, the use of vancomycin is increasing [3, 4].

Vancomycin is the commonly used glycopeptide to treat aminopenicillin-resistant gram-positive bacteria [5]. Because it has adverse effects such as nephrotoxicity, studies have examined the pharmacokinetics of vancomycin to minimize its toxicity and maximize its therapeutic effect [6]. The guidelines for vancomycin dosing strategies to assist clinicians and pharmacists have been revised. By attempting to implement therapeutic monitoring using current knowledge of vancomycin pharmacodynamics and in view of potential problems with efficacy, resistance, and toxicity, these guidelines are intended to improve patient outcomes [7, 8]. Vancomycin is the drug of choice for methicillin-resistant *Staphylococcus aureus* (MRSA) infections [8, 9]. The pharmacodynamic parameter that best predicts vancomycin efficacy in invasive MRSA infections is the ratio of the 24 h area under the concentration-time curve to minimum inhibitory concentration (AUC_24_/MIC) [10]. Serum vancomycin trough concentrations have been used as a surrogate marker for identifying an AUC_24_/MIC of ≥ 400 [11], although several recent studies found that the trough concentration poorly reflects the AUC_24_/MIC target and that a high trough concentration increases the risk of nephrotoxicity [8, 12].

To our knowledge, few studies have examined the target vancomycin concentration in the treatment of non-MRSA gram-positive infections, especially those caused by *Enterococcus* species. Therefore, we investigated whether vancomycin concentration targets such as the trough level and AUC/MIC are associated with the treatment outcome in patients with bacteremia caused by *Enterococcus* species.

## Methods

### Study population and design

A retrospective, single-center, cohort study was conducted at Severance Hospital, a 2400-bed, tertiary-care hospital in Seoul, South Korea. The electronic medical records of patients > 18 years of age with enterococcal bacteremia treated with vancomycin between January 1, 2007 and December 31, 2017 were reviewed. The age range of patients enrolled in this study was from 26 to 91 years. The exclusion criteria were as follows: presence of polymicrobial bacteremia, isolates resistant to vancomycin, missing trough vancomycin concentration, and vancomycin MIC data. Data collected from the patients’ medical records included demographic characteristics, comorbidities, source of bacteremia, antimicrobial treatment data, duration of bacteremia, relapse of bacteremia, and mortality. The primary outcome was 28-day all-cause mortality. The study was approved by the Institutional Review Board (IRB) of the Yonsei University Health System Clinical Trial Center (4-2020-0161). All methods were carried out in accordance with relevant guidelines and regulations under Ethics approval. Since the study was retrospective and the study subjects were anonymized, the IRB waived the requirement for written informed consent from the patients.

### Definitions

Bacteremia was defined as at least one positive blood culture with an identifiable source and clinical manifestations consistent with bacteremia, or at least two separate blood cultures positive for *Enterococcus* species. Persistent blood stream infection was defined as the case when the bacteria were continuously identified in two or more follow-up cultures after the first positive *Enterococcus* blood culture [13]. For patients with more than one episode of *Enterococcus* bacteremia, only the first episode during the study period was included. The duration of bacteremia was defined as the number of days from the first positive blood culture to the date of the first negative *Enterococcus* blood culture. Relapse occurred if the cultures became negative for more than 2 days and then became positive within 90 days. Vancomycin-induced nephropathy (VIN) was defined as an increase in the serum creatinine level of ≥ 0.5 mg/dL, or a 50% increase from baseline in consecutive daily readings, or a decrease in the calculated creatinine level of 50% from baseline on 2 consecutive days in the absence of an alternative explanation [6]. The inappropriate empirical antibiotics use means that the empirical antibiotics used before identification of the bacteria is not suitable for the drug susceptibility test result. Community-acquired bacteremia is defined as bacteremia occurred within 48 h of hospitalization. And hospital-acquired bacteremia is a bacteremia occurred 48 h after admission.

### Microbiological data

The species in the clinical isolates were identified using the VITEK®2 system with GNI cards (bioMérieux, Marcy-l’Étoile, France) until 2014 or Microflex MALDI-TOF mass spectrometry with Biotyper software 3.1 (Bruker Daltonics, Leipzig, Germany) after clinical adoption in 2014. Antimicrobial susceptibility tests were performed using the disk diffusion method or a VITEK-2 N131 card (bioMérieux). The results were interpreted according to the Clinical and Laboratory Standards Institute guidelines [14].

### Vancomycin dosing and pharmacodynamics data

To obtain trough concentrations, blood samples were collected immediately before the next vancomycin dose. Samples for peak concentrations were collected 1 h after intravenous vancomycin infusion was completed. Based on the hospital guidelines, all venous blood samples used to determine steady-state serum concentrations were obtained after administering at least four doses of vancomycin. The serum vancomycin concentrations were analyzed with a chemiluminescence microparticle immunoassay using an Architect automated immunochemistry analyzer (Abbott Labs, Chicago, IL, USA). All pharmacokinetic calculations and modeling were performed using the MW/Pharm software package (ver. 3.82; Mediware, Zuidhorn, the Netherlands). And the trough level used to categorize in this study was measured through the first blood samples after at least four consecutive doses of vancomycin.

Demographic parameters, including weight, height, ethnicity, and sex, were imported from the hospital’s electronic records. Renal function was estimated using the most recent serum creatinine level. Estimated curves were fitted using the default settings of the posterior Bayesian estimation after the simulation. The area under the curve (AUC) of the estimated vancomycin concentration was calculated in a chronological manner. Patients on renal dialysis were screened for additional creatinine concentrations.

### Statistical analysis

The relationships between the vancomycin trough concentration or the AUC_24_/MIC and mortality in patients with *E*. *faecium* or *E. faecalis* bacteremia were analyzed using both continuous and categorical variables. The Kolmogorov-Smirnov test and Shapiro-Wilk test were conducted to verify the normality of the continuous variables. The independent *t*-test and the Mann–Whitney test were used to compare the continuous variables of the two groups. The chi-square test or Fisher’s exact test were used to compare categorical variables. Potentially significant variables identified in the univariate analysis were included in a multivariate logistic regression analysis to identify the risk factors associated with 28-day mortality. The Kaplan–Meier survival curve was used to compare mortality according to vancomycin trough concentrations. All statistical analyses were performed using SPSS Statistics ver. 25.0 (IBM Corp., Armonk, NY, USA).

## Results

A total of 209 patients had confirmed enterococcal bacteremia. Of these, 73 patients had polymicrobial bacteremia, 62 had vancomycin-resistant enterococci, and 37 did not perform therapeutic drug monitoring (TDM). Finally, 37 patients (22 men, 15 women; mean age, 60.4 years) were enrolled: 26 cases of *E*. *faecium* and 11 of *E. faecalis*. (Additional file 1: Table S1). The most common comorbidities were solid cancer (56.8%), followed by hypertension (40.5%), and diabetes mellitus (35.1%). The most common source of infection was biliary infection (40.5%), followed by peritonitis (21.6%).

**Table 1:** Baseline demographic and clinical features of patients with enterococcal bacteremia

		28-day all mortality		
**Characteristics**	Total (n = 37)	Survivors (n = 29)	Non-survivors (n = 8)	*P *value
**Demographics**				
Age (year, mean±SD)	60.5 ± 13.2	62.8 ± 2.3	52.3 ± 4.4	0.044
BMI (mean±SD)	21.4 ± 3.1	21.2 ± 0.6	22.4 ± 1.1	0.323
Male (%)	22 (59.5)	18(62.1)	4(50.0)	0.412
Community AB (%)	9 (24.3)	7 (24.1)	2 (25.0)	0.643
Hospital AB (%)	28 (75.7)	22 (75.9)	6 (75.0)	0.643
**Comorbidities** (%)				
Solid cancer	21 (56.8)	17 (58.6)	4 (50.0)	0.483
HTN	15 (40.5)	11 (37.9)	4 (50.0)	0.412
DM	13 (35.1)	11 (37.9)	2 (25.0)	0.408
Chronic liver disease	11 (29.7)	8 (27.6)	3 (37.5)	0.444
Organ transplantation	9 (24.3)	7 (24.1)	2 (25.0)	0.643
Chronic renal disease	9 (24.3)	6 (20.7)	3 (37.5)	0.292
Cerebrovascular disease	7 (18.9)	6 (20.7)	1 (12.5)	0.521
Cardiovascular disease	6 (16.2)	5 (17.2)	1 (12.5)	0.613
Hematologic malignancies	4 (10.8)	3 (10.3)	1 (12.5)	0.640
CHF	3 (8.1)	2 (6.9)	1 (12.5)	0.530
Hemiplegia	2 (5.4)	2 (6.9)	0 (0.0)	0.610
PAOD	1 (2.7)	0 (0.0)	1 (12.5)	0.216
ILD	1 (2.7)	1 (3.4)	0 (0.0)	0.784
Antibiotic use in 30 day (%)	27 (73.0)	22 (75.9)	5 (62.5)	0.367
Steroid use in 30 day (%)	16 (43.2)	12 (41.4)	4 (50.0)	0.483
Anticancer drug use in 30 day (%)	9 (24.3)	7 (24.1)	2 (25.0)	0.643
Immunosuppressant use in 30 day (%)	9 (24.3)	7 (24.1)	2 (25.0)	0.643
Vancomycin induced AKI (%)	6 (16.2)	4 (13.8)	2 (25.0)	0.591
Septic shock (%)	8 (21.6)	4 (13.8)	4 (50.0)	0.049
**Source of bacteremia** (%)				
Biliary	15 (40.5)	12 (41.4)	3 (37.5)	0.588
Peritonitis	8 (21.6)	7 (24.1)	1 (12.5)	0.435
UTI	1 (2.7)	0 (0.0)	1 (12.5)	0.216
Skin	1 (2.7)	1 (3.4)	0 (0.0)	0.784
CRBSI	1 (2.7)	1 (3.4)	0 (0.0)	0.784
Foreign device	1 (2.7)	1 (3.4)	0 (0.0)	0.784
Others	2 (5.4)	1 (3.4)	1 (12.5)	0.390
Primary	8 (21.6)	6 (20.7)	2 (25.0)	0.565
ICU stay (%)	14 (37.8)	10 (34.5)	4 (50.0)	0.343
SOFA score (mean±SD)	7.0 ± 4.8	6.2 ± 0.8	9.9 ± 1.9	0.056
Persistent BSI (%)	18 (48.6)	15 (51.7)	3 (37.5)	0.379
Recurrence of same BSI (%)	5 (13.5)	5 (17.2)	0 (0.0)	0.272
Initial empirical inappropriate antibiotics (%)	16 (43.2)	13 (44.8)	3 (37.5)	0.517
Combination therapy (%)	8 (21.6)	6 (20.7)	2 (25.0)	0.565
**PK/PD parameter** (%)				
Trough level ≤15 µg/mL	19 (51.4)	12 (41.4)	7 (87.5)	
Trough level>15 µg/mL	18 (48.6)	17 (58.6)	1 (12.5)	0.042
AUC_24_/MIC≤389	7 (18.9)	5 (17.2)	2 (25.0)	
AUC_24_/MIC>389	30 (81.1)	24 (82.8)	6 (75.0)	0.479
BMI, body mass index; Community AB, Community acquired bacteremia; Hospital AB, Hospital acquired bacteremia; HTN, hypertension; DM, diabetes mellitus; CHF, congestive heart failure; PAOD peripheral arterial occlusive disease; ILD, interstitial lung disease; AKI. Acute kidney disease, UTI, urinary tract infection; CRBSI, catheter-related blood stream infection; BSI, blood stream infection, AUC, area under curve; MIC minimum inhibitory concentration; Other cases of bacteremia include infective endocarditis Continuous variables are shown as the mean ± standard deviation (SD) and categorical variables, as numbers (percentage)				

The 28-day mortality rate was 21.6% (8/37). No significant differences were found in the baseline characteristics, except for age (Table 1). Vancomycin-induced nephropathy occurred in 6 of the 37 patients. In the MIC distributions for vancomycin, there were 3 cases of MIC 2 mg/L, 18 cases of MIC 1 mg/L, and 16 cases of MIC < 0.5 mg/L. The median value of AUC_24_ in this study was 474.21, the minimum value was 181.17, and the maximum was 1111.41.

**Fig. 1:**
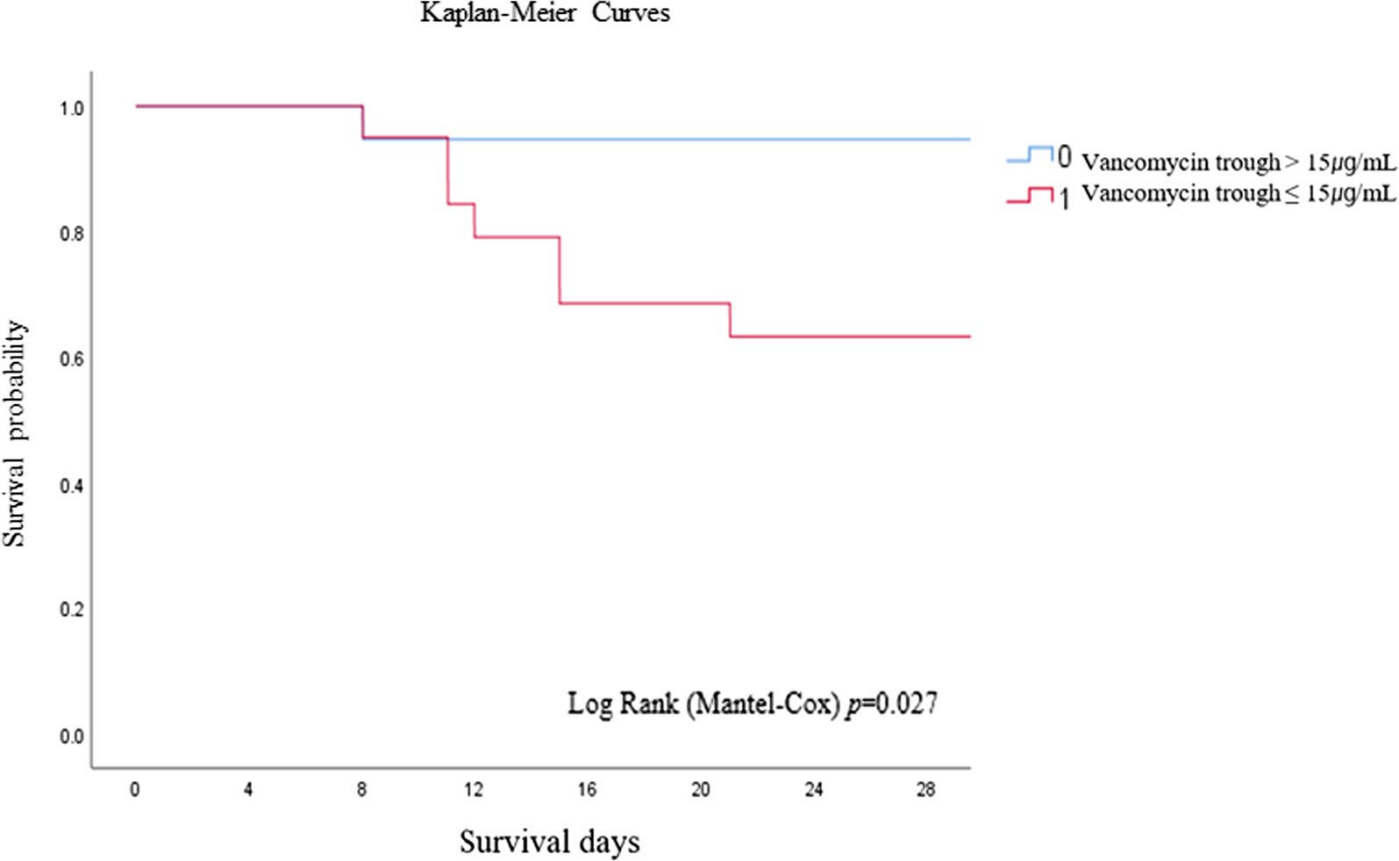
Kaplan-Meier survival analysis of patients according to vancomycin trough level

In univariate analyses, vancomycin trough concentration (≤ 15 µg/mL) (*p* = 0.042), age (*p* = 0.044), and septic shock (*p* = 0.049) were associated with 28-day mortality. The proportion of patients achieving AUC_24_/MIC ≤ 389 did not differ between the two groups (*p* = 0.479). In a multivariate analysis, vancomycin trough concentration (≤15 µg/mL) (*p* = 0.041; odds ratio (OR), 119.013; 95% confidence interval (CI), [1.207–11732.690]), and younger age (*p* = 0.031; OR, 1.212; 95% CI, [1.018–1.442]) were associated with mortality in patients with enterococcal bacteremia (Table 2). Moreover, in the Kaplan–Meier analysis, the group with vancomycin trough levels of 15 µg/mL or lower had a higher 28-day mortality (log rank = 0.027) (Fig. 1).

**Table 2:** Multivariate logistic regression analysis for factors associated with 28-day mortality

Variables	OR	95 % CI	*p* value
Younger age	1.212	1.018~1.442	0.031
Vancomycin trough level≤15 µg/mL	119.013	1.207~11732.690	0.041
Septic shock	18.369	0.997~338.574	0.050

OR, odds ratio; CI, confidence interval

## Discussion

In this study, we found that vancomycin trough concentration (≤ 15 µg/mL) was significantly associated with increased mortality in patients with *Enterococcus* bacteremia.

Many studies have examined pharmacokinetic/pharmacodynamic (PK/PD) parameters to maintain the efficiency of vancomycin and to minimize its adverse effects, but most of these studies have examined MRSA infection. Of the PK/PD parameters, the trough level and AUC_24_/MIC have been reported to describe the effectiveness of vancomycin [15, 16]. The clinical success and renal toxicity of vancomycin treatment are exposure-dependent and characterized by the AUC_24_. The exact range targeted by clinicians is influenced by the AUC_24_ estimation and bacterial MIC [15, 17]. Using AUC-based vancomycin therapeutic drug monitoring (TDM) helps to individualize the estimation of the AUC_24_/MIC and minimize the risk of toxicity due to unnecessary overexposure.

Although trough concentration is a known surrogate marker for AUC_24_/MIC, the method using only trough-based vancomycin TDM is controversial for several reasons. First, the vancomycin trough concentration poorly characterizes the AUC_24_, and adequate vancomycin AUC_24_ levels can be obtained at trough concentrations < 15 mg/L. Vancomycin-associated nephrotoxicity also increases when the vancomycin trough concentration is >15 mg/L. These factors provide evidence for an AUC-based approach to vancomycin TDM [18]. Thus, according to the Infectious Diseases Society of America guidelines, the trough level does not correlate well with the AUC, and trough-only monitoring with a target of 15–20 µg/mL is no longer recommended based on its efficacy in patients with MRSA infections [10]. However, because it may be difficult to determine the AUC_24_/MIC in a clinical setting, trough serum concentrations are still monitored clinically. Moreover, there is insufficient evidence to recommend whether trough level-only or AUC/MIC-guided vancomycin monitoring should be used for patients with non-MRSA infections.

In one study of PK/PD determinants of vancomycin efficacy in enterococcal bacteremia, a vancomycin AUC/MIC ≥ 389 achieved within 72 h was associated with reduced mortality. However, the study found that vancomycin trough concentrations differed significantly between survivors and non-survivors [19]. According to Nakamura et al. [20] neither the AUC_24_/MIC nor the trough concentration of vancomycin is significantly associated with mortality in patients with *E*. *faecium* bacteremia. The trough concentration was higher in non-survivors than in survivors, and AUC_24_/MIC did not differ significantly between non-survivors and survivors.

In our study, vancomycin trough levels of 15 µg/mL or lower were associated with mortality in patients with enterococcal bacteremia, but the AUC_24_/MIC was not. Zelenitsky et al. reported that the survival rate was higher when the vancomycin trough concentration was maintained above 15 mg/L than when low trough concentrations were maintained [21]. In addition, Kullar et al. reported that the vancomycin treatment period was significantly shorter in the group in which the vancomycin trough concentration was higher than the group to which the vancomycin trough concentration was applied at 5 to 20 mg/L, and the rate at which the bacteria were negatively converted was also found to be significantly higher in the group that maintained the higher trough concentration [22]. According to a meta-analysis comparing high and low vancomycin serum trough regimen groups with gram-positive bacterial infections, there was no significant difference between the two in all-cause mortality or risk of clinical failure; however, a subgroup analysis confirmed that treatment failure was reduced in high vancomycin trough regimen groups [23]. Therefore, in enterococcal bacteremia, the trough level may be a good parameter for monitoring the therapeutic effects of vancomycin.

All enterococcal BSIs treated with vancomycin were included in this study to examine the therapeutic effect of vancomycin on Enterococcus species. Therefore, strains susceptible to ampicillin were added in this study. In most cases, vancomycin was used for ampicillin-susceptible Enterococcus species when beta-lactam antibiotics could not be used due to its side effects, or was used without checking the results of antimicrobial susceptibility tests. Ampicillin is the preferred antibiotic used to treat ampicillin-sensitive enterococcal infections. However, according to several studies comparing the therapeutic effects of beta-lactam antibiotics and vancomycin on ampicillin-susceptible enterococcal BSI, there was no significant difference in mortality [24]. Therefore, when needed, or when beta-lactam antibiotics are difficult to use, vancomycin can be considered as a treatment option for ampicillin-susceptible strains.

An important concern when using vancomycin is the occurrence of acute kidney injury. A higher vancomycin trough concentration increases the risk of VIN [20, 25]. Although most vancomycin-induced nephrotoxic events are reversible, many studies support there is increased mortality from AKI of any cause, including vancomycin-induced nephropathy [26]. While 6 of our 37 patients developed VIN, it was not statistically related to trough concentration (*p* = 0.09) or 28-day mortality. (*p*= 0.446).

In this study, mortality was higher at younger ages. Aging process causes structural and functional changes in multiple organ systems, especially the kidneys, and is known to affect PK/PD of drugs by causing changes in body composition, drug absorption, distribution, and clearance [27]. These changes may have affected the patient’s prognosis or treatment outcome.

This study has several limitations that are inherent to its retrospective design. As with any observational study, there remains a possibility that unmeasured confounders influenced our findings. According to several studies, it is well known that sepsis or septic shock affects drug absorption, distribution and other pharmacological processes due to pathophysiological changes such as decreased perfusion to body organs, interstitial edema or increased capillary permeability [28]. Therefore, the PK/PD alterations may not have been reflected depending on the patients’ disease severity. In addition, in the early stage of sepsis, extravascular volume expansion with fluid loading and capillary leak causes a change in volume distribution, which can cause hyperfiltration in the kidney and lead to an alteration of drug concentration. Thereby, it may have affected the prognosis of non-survivors with low trough levels. For these reasons, we cannot exclude the possibility that residual bias affected our findings. Another limitation of this study was that only patients with bacteremia were enrolled, and patients with non-bacteremic sepsis were not analyzed. In addition, the single-center nature of a referral tertiary hospital leads to selection bias in severe cases admitted to our hospital. A multicenter study with a larger sample size is needed.

## Conclusions

In conclusion, a vancomycin trough level of 15 µg/mL or lower was significantly associated with 28-day mortality in patients with enterococcal bacteremia. However, larger prospective studies are needed to examine the association of vancomycin PK/PD parameters for treating other gram-positive pathogens, especially *Enterococcus* species.

## Supplementary Information

**Additional file 1: Table S1.** Baseline characteristics and clinical features according to Enterococcus species in patients with enterococcal bacteremia.

## Data Availability

All datasets generated during the current study are available from the corresponding author on reasonable request.

## Abbreviations

AUC: Area under the concentration-time curve
MIC: Minimal inhibitory concentration
PK: Pharmacokinetics
PD: Pharmacodynamics
TDM: Therapeutic drug monitoring
VIN: Vancomycin-induced nephropathy
