# Supplementary material for: Association of vancomycin trough concentration on the treatment outcome of patients with bacteremia caused by Enterococcus species
Source: BMC Infect Dis. 2021 Oct 26;21:1099. doi: 10.1186/s12879-021-06809-x (PMC8547083; doi:10.1186/s12879-021-06809-x)
Supplement: Supplementary file 1 — Additional file 1: Table S1. Baseline characteristics and clinical features according to Enterococcus species in patients with enterococcal bacteremia. [file 12879_2021_6809_MOESM1_ESM.docx]

**table S1. Baseline characteristics and clinical features according to Enterococcus species in patients with enterococcal bacteremia**

|  | 28 day all-mortality | | | | | | | | |
| --- | --- | --- | --- | --- | --- | --- | --- | --- | --- |
|  | | *Enterococcus faecium* | | | | *Enterococcus faecalis* | | | |
| **Characteristics** | | Total (n=26) | Survivors (n=20) | Non-survivors (n=6) | *P* value | Total (n=11) | Survivors (n=9) | Non-survivors (n=2) | *P* value |
| **Demographics** | |  |  |  |  |  |  |  |  |
| Age (year, mean±SD) | | 58.73 ± 8.5 | 59.75 ± 1.7 | 55.33 ± 4.6 | .294 | 64.64 ± 20.4 | 69.44 ± 6.2 | 43.00 ± 11.0 | .878 |
| BMI (mean±SD) | | 21.62 ± 3.3 | 21.19 ± 0.7 | 23.07 ± 1.3 | .981 | 20.99 ± 2.9 | 21.11 ± 1.0 | 20.45 ± 1.8 | .331 |
|  | |  |  |  |  |  |  |  |  |
| Male (%) | | 15 (57.7) | 12 (60.0) | 3 (50.0) | .664 | 7 (63.6) | 6 (66.7) | 1 (50.0) | .658 |
|  | |  |  |  |  |  |  |  |  |
| Community AB (%) | | 3 (11.5) | 3 (15.0) | 0 (0.0) | .313 | 6 (54.5) | 4 (44.4) | 2 (100) | .154 |
| Hospital AB (%) | | 23 (88.5) | 17 (85.0) | 6 (100.0) | .313 | 5 (45.5) | 5 (55.6) | 0 (0.0) | .154 |
|  | |  |  |  |  |  |  |  |  |
| Ampicillin susceptible strain | | 4 (15.4) | 4 (20.0) | 0 (0.0) | .234 | 11 (100.0) | 9 (100.0) | 2 (100.0) | - |
|  | |  |  |  |  |  |  |  |  |
| **Comorbidities** (%) | |  |  |  |  |  |  |  |  |
| Solid cancer | | 18 (69.2) | 15 (75.0) | 3 (50.0) | .245 | 3 (27.3) | 2 (22.2) | 1 (50.0) | .425 |
| HTN | | 9 (34.6) | 6 (30.0) | 3 (50.0) | .366 | 6 (54.5) | 5 (55.6) | 1 (50.0) | .887 |
| DM | | 9 (34.6) | 7 (35.0) | 2 (33.3) | .940 | 4 (36.4) | 4 (44.4) | 0 (0.0) | .237 |
| Chronic liver disease | | 9 (34.6) | 7 (35.0) | 2 (33.3) | .940 | 2 (18.2) | 1 (11.1) | 1 (50.0) | .197 |
| Organ transplantation | | 7 (26.9) | 5 (25.0) | 2 (33.3) | .686 | 2 (18.2) | 2 (22.2) | 0 (0.0) | .461 |
| Chronic renal disease | | 3 (11.5) | 1 (5.0) | 2 (33.3) | .057 | 6 (54.5) | 5 (55.6) | 1 (50.0) | .887 |
| Hematologic malignancies | | 3 (11.5) | 2 (10.0) | 1 (16.7) | .654 | 1 (9.1) | 1 (11.1) | 0 (0.0) | .621 |
| Cerebrovascular disease | | 2 (7.7) | 2 (10.0) | 0 (0.0) | .420 | 5 (54.5) | 4 (44.4) | 1 (50.0) | .887 |
| Cardiovascular disease | | 2 (7.7) | 1 (5.0) | 1 (16.7) | .347 | 4 (36.4) | 4 (44.4) | 0 (0.0) | .237 |
| ILD | | 1 (3.8) | 1 (5.0) | 0 (0.0) | .576 | 0 (0.0) | 0 (0.0) | 0 (0.0) | - |
| CHF | | 1 (3.8) | 0 (0.0) | 1 (16.7) | .063 | 2 (18.2) | 2 (22.2) | 0 (0.0) | .461 |
| PAOD | | 1 (3.8) | 0 (0.0) | 1 (16.7) | .063 | 0 (0.0) | 0 (0.0) | 0 (0.0) | - |
| Hemiplegia | | 0 (0.0) | 0 (0.0) | 0 (0.0) | - | 2 (18.2) | 2 (22.2) | 0 (0.0) | .461 |
|  | |  |  |  |  |  |  |  |  |
| Antibiotic use in 30 day (%) | | 22 (84.6) | 17 (85.0) | 5 (83.3) | .921 | 5 (45.5) | 5 (55.6) | 0 (0.0) | .154 |
| Steroid use in 30 day (%) | | 13 (50.0) | 9 (45.0) | 4 (66.7) | .352 | 3 (27.3) | 3 (33.3) | 0 (0.0) | .338 |
| Anticancer drug use in 30day (%) | | 9 (34.6) | 7 (35.0) | 2 (33.3) | .940 |  |  |  |  |
| Immunosuppressant use in 30 day (%) | | 7 (26.9) | 5 (25.0) | 2 (33.3) | .686 | 2 (18.2) | 2 (22.2) | 0 (0.0) | .461 |
|  | |  |  |  |  |  |  |  |  |
| Vancomycin induced AKI (%) | | 3 (11.5) | 2 (10.0) | 1 (16.7) | .654 | 3 (27.3) | 2 (22.2) | 1 (50.0) | .425 |
| Septic shock (%) | | 7 (26.9) | 4 (20.0) | 3 (50.0) | .146 | 1 (9.1) | 0 (0.0) | 1 (50.0) | .026 |
| **Source of bacteremia** (%) | |  |  |  |  |  |  |  |  |
| Biliary | | 13 (50.0) | 10 (50.0) | 3 (50.0) | 1.000 | 2 (18.2) | 2 (22.2) | 0 (0.0) | .461 |
| Peritonitis | | 8 (30.8) | 7 (35.0) | 1 (16.7) | .393 | 0 (0.0) | 0 (0.0) | 0 (0.0) | - |
| Primary | | 4 (15.4) | 2 (10.0) | 2 (33.3) | .165 | 4 (36.4) | 4 (44.4) | 0 (0.0) | .237 |
| CRBSI | | 1 (3.8) | 1 (5.0) | 0 (0.0) | .576 | 0 (0.0) | 0 (0.0) | 0 (0.0) | - |
| UTI | | 0 (0.0) | 0 (0.0) | 0 (0.0) | - | 1 (9.1) | 0 (0.0) | 1 (50.0) | .026 |
| Skin | | 0 (0.0) | 0 (0.0) | 0 (0.0) | - | 1 (9.1) | 1 (11.1) | 0 (0.0) | .621 |
| Foreign device | | 0 (0.0) | 0 (0.0) | 0 (0.0) | - | 1 (9.1) | 1 (11.1) | 0 (0.0) | .621 |
|  | |  |  |  |  |  |  |  |  |
| ICU stay (%) | | 9 (34.6) | 6 (30.0) | 3 (50.0) | .366 | 5 (45.5) | 4 (44.4) | 1 (50.0) | .887 |
| SOFA score (mean±SD) | | 7.08 ± 5.0 | 6.25 ± 1.1 | 9.83 ± 2.0 | .701 | 6.91 ± 4.5 | 6.22 ± 1.2 | 10.00 ± 6.0 | .090 |
|  | |  |  |  |  |  |  |  |  |
| Persistent BSI (%) | | 13 (50.0) | 10 (50.0) | 3 (50.0) | 1.000 | 5 (45.5) | 5 (55.6) | 0 (0.0) | .154 |
| Recurrence of same BSI (%) | | 5 (19.2) | 5 (25.0) | 0 (0.0) | .173 | 0 (0.0) | 0 (0.0) | 0 (0.0) | - |
| Initial empirical inappropriate antibiotics (%) | | 11 (42.3) | 9 (45.0) | 2 (33.3) | .612 | 5 (45.5) | 4 (44.4) | 1 (50.0) | .887 |
|  | |  |  |  |  |  |  |  |  |
| **PK/PD parameter** (%) | |  |  |  |  |  |  |  |  |
| Trough level ≤15μg/mL | | 16 (61.5) | 10 (50.0) | 6 (100.0) |  | 3 (27.3) | 2 (22.2) | 1 (50.0) |  |
| Trough level>15μg/mL | | 10 (38.5) | 10 (50.0) | 0 (0.0) | .027 | 8 (72.7) | 7 (77.8) | 1 (50.0) | .425 |
| AUC_24_/MIC≤389 | | 3 (11.5) | 2 (10.0) | 1 (16.7) |  | 4 (36.4) | 3 (33.3) | 1 (50.0) |  |
| AUC_24_/MIC>389 | | 23 (88.5) | 18 (90.0) | 5 (83.3) | .654 | 7 (63.6) | 6 (81.8) | 1 (50.0) | .658 |
